# Gorlin-Goltz Syndrome: Case Report of a Rare Hereditary Disorder

**DOI:** 10.1155/2012/475439

**Published:** 2012-09-23

**Authors:** Ashutosh Agrawal, Aditi Murari, Sunil Vutukuri, Arun Singh

**Affiliations:** ^1^Department of Oral Pathology and Microbiology, Institute of Dental Sciences, Uttar Pradesh, Bareilly 243006, India; ^2^Department of Oral Pathology and Microbiology, Padmashree Dr. D. Y. Patil Dental College and Hospital, Nerul, Maharashtra, Navi Mumbai 400706, India; ^3^Department of Oral Pathology and Microbiology, Kothiwal Dental College and Research Centre, Uttar Pradesh, Moradabad 244001, India

## Abstract

*Introduction*. Gorlin-Goltz syndrome is an inherited autosomal dominant disorder with complete penetrance and extreme variable expressivity. *Case Report*. The present paper highlights the importance of diagnostic criteria and histopathology in early and prompt diagnosis which will lead to proper treatment and genetic counseling of the patient. *Discussion*. Gorlin-Goltz syndrome is about multisystem process comprising the triad of basal cell nevi, jaw keratocysts, and skeletal anomalies. A spectrum of other neurological, ophthalmic, endocrine and genital manifestations is known to be variably associated with this triad. Diagnosis of the syndrome is based on major and minor criteria. *Conclusion*. This paper emphasizes the importance of oral and maxillofacial health professionals in the early diagnosis of nevoid basal cell carcinoma syndrome and in a preventive multidisciplinary approach to provide a better prognosis to the patient.

## 1. Introduction

Gorlin-Goltz syndrome is an infrequent multisystemic disease that is inherited in a dominant autosomal way, which shows a high level of penetrance and variable expressiveness. In 1894, Jarisch and White made the first descriptions of patients with this syndrome, highlighting the presence of multiple basocellular carcinomas. Nevertheless, it was not until 1960 when Gorlin and Goltz established a classical triad that characterises the diagnosis of this syndrome (multiple basocellular epitheliomas, keratocysts in the jaws, and bifid ribs) [1]. A spectrum of other neurological, ophthalmic, endocrine, and genital manifestations are known to be variably associated with this triad. The incidence of this syndrome is estimated to be 1 in 50,000 to 150,000 in general population, but perceived incidence may vary by region. Males and females are equally affected [2].

## 2. Case Report

**Figure 1:**
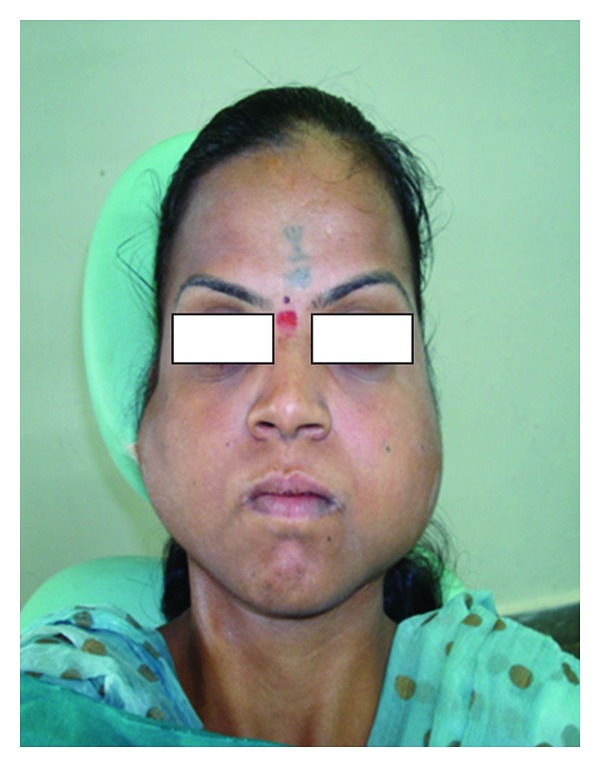
The figure shows frontal bossing, broad nasal bridge, hypertelorism, and mandibular prognathism.

A female patient 25 years old come to our department with chief complaint of swelling in bilateral cheeks. The duration of the swelling was 10 month and the growth was slow in nature. She gave a medical history of similar bilateral swellings when she was 12 years old and had underwent surgery for the same. On examination, the swelling was firm and slightly tender on right side. Examination of the face showed frontal bossing, broad nasal bridge, hypertelorism, and mandibular prognathism (Figure 1).

**Figure 2:**
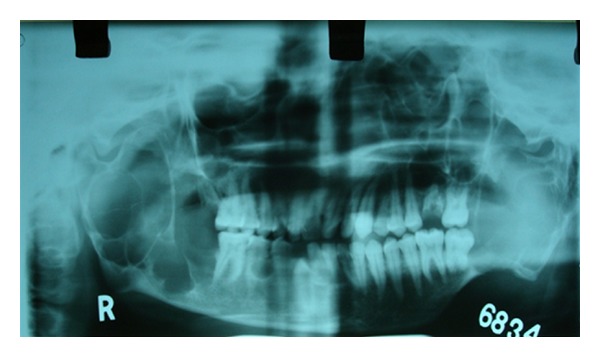
Orthopantomograph showing multiple multilocular well-defined radiolucencies with sclerotic border located in maxilla and mandible.

Orthopantomograph revealed multiple multilocular well-defined radiolucencies with sclerotic border located in maxilla and mandible (Figure 2). 

The presence of multiple cysts in the jaws and extraoral examination raised a suspicion of Gorlin syndrome and so other relevant investigations were done.

**Figure 3:**
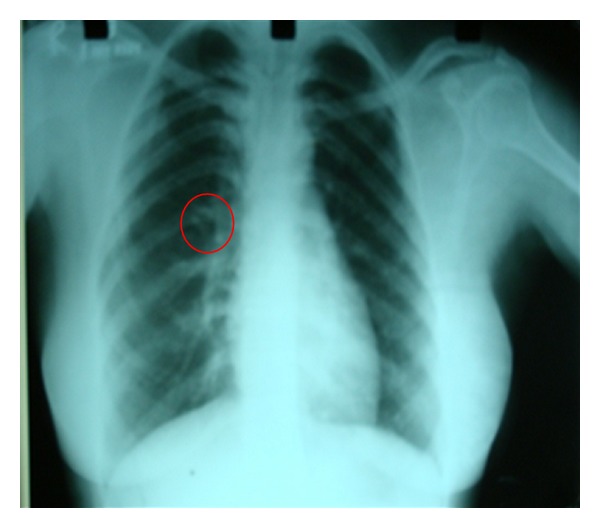
Chest radiograph showing a bifid right fifth rib.

Chest radiograph showed a bifid fifth rib (Figure 3). The reports of the patient also revealed the presence of nabothian cyst in cervix.

**Figure 4:**
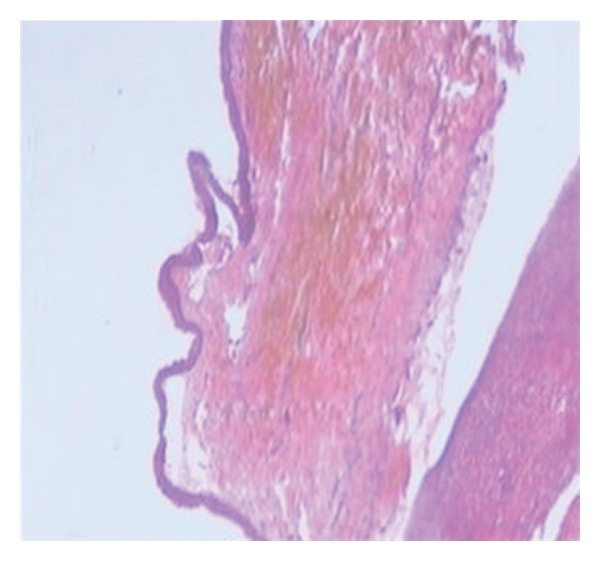
H&E stained section under scanner view shows flat epithelial-connective tissue interface, detachment of epithelial lining due to inflammation and folded epithelial lining.

**Figure 5:**
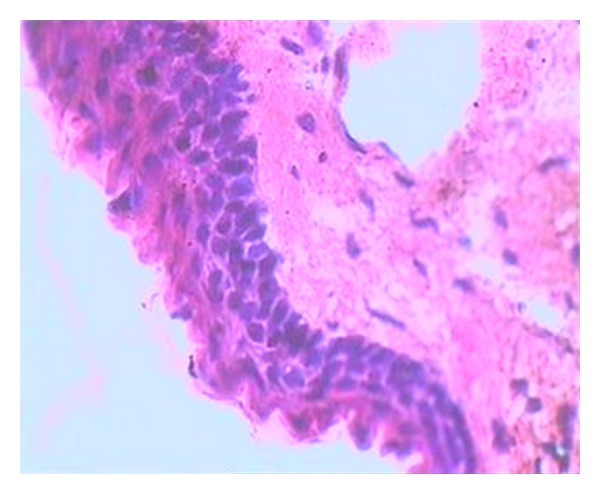
H&E stained section under 40X magnification shows 6–8 layers thick epithelial lining which is parakeratinized and corrugated.

An incisional biopsy of the swelling in left and right side of mandible was advised. Histopathological examination of specimen revealed parakeratinised stratified squamous epithelium with palisading pattern of columnar cells along with keratin flakes with few giant cells and inflammatory cells (found in the specimen of the left side of the mandible) suggestive of odontogenic keratocyst on the right side and an odontogenic keratocyst with secondary infection on the left side (Figures 4 and 5). Surgical enucleation of the cysts was performed in the department of oral surgery. Any skin lesions like basal cell nevus or keratosis were not seen in the patient. Thorough clinical and radiological examination were done for the parents of the patient but they showed no signs of Gorlin-Goltz syndrome.

Based on the clinical, radiographic, and histologic findings, and referring to the diagnostic criteria for nevoid BCC syndrome established by Evanset al. [4], and modified by Kimonos et al. in 1997 [5], the patient was diagnosed as having Gorlin-Goltz syndrome. 

## 3. Discussion

The Gorlin-Goltz syndrome is an autosomal dominant inherited syndrome manifested by multiple defects involving the skin, nervous system, eyes, endocrine system, and bones. It is also known as basal cell nevus syndrome, multiple basal cell carcinoma syndrome, Gorlin syndrome, or hereditary cutaneomandibular polyonocosis, multiple nevoid basal cell epithelioma-jaw cysts, or bifid rib syndrome [3]. The diagnostic criteria for nevoid BCC was established by Evans et al. [4], and modified by Kimonis et al. in 1973 [5, 6]. According to them diagnosis of Gorlin-Goltz syndrome can be established when two major or one major and two minor are present which are described below.

**Major Criteria are as follows:**

1. more than 2 BCCs or one under age of 20 year,
2. odontogenic keratocyst,
3. three or more palmar pits,
4. bilamellar calcification of falx cerebri,
5. bifid, fused, or splayed ribs,
6. first-degree relative with NBCCS.

**Minor Criteria are as follows:**

1. macrocephaly adjusted for height,
2. fontal bossing, cleft lip/palate, and hypertelorism,
3. sprengel deformity, pectus, and syndactyly of digits,
4. bridging of sella turcica, hemivertebrae, and flame-shaped radiolucencies,
5. ovarian fibroma,
6. medulloblastoma [6, 7].

**Odontogenic Keratocysts**

Woolgar et al. [8] and Dominiguez et al. [9] found significant differences between syndrome keratocysts and single keratocysts. Syndrome keratocysts were found to have a markedly increased number of satellite cysts, solid islands of epithelial proliferation, odontogenic rests within the capsule, and mitotic figures in the epithelial lining of the main cavity.

There are immunochemical differences between syndromal and solitary keratocysts. Woolgar et al. noted that syndrome keratocysts tend to occur at a much earlier age than single keratocysts [7, 8].

Less than 10% of patients with multiple OKCs have other manifestations of this syndrome; however, it has been suggested that multiple OKCs alone may be the confirmatory of the syndrome. Two types of keratocysts have been distinguished based on differences in the histology and behavior: the more common parakeratotic odontogenic keratocyst (P-OKC) and the less common orthokeratotic odontogenic keratocyst (O-OKC). First, the P-OKC has a more aggressive growth potential and a higher recurrence rate than the O-OKC and other odontogenic cysts. Second, in a minority of patients (particularly, young patients with multiple cysts), the P-OKC is a part of the Jaw cyst-Basal cell nevus-Bifid rib syndrome. Although benign, the recurrence rate of P-OKC is high, ranging from 12% to 62.5% [10].

For apparently isolated cases, detailed examination and X-ray investigation of the relatives should be undertaken before concluding.It is particularly helpful to follow a specific clinical protocol in the examination of these subjects [11] (Table 1).

## 4. Conclusion

To summarize,it can be said that Gorlin-Goltz syndrome is a dominant autosomal genetic process, which is of particular interest to the oral and maxillofacial health experts. The importance of recognition of this syndrome is because of its malignant potential.In order to be able to establish early diagnosis of NBCCS, specialists should carry out clinical and imaging examinations in early ages of life. The fact that its transmission is autosomal dominant with good penetrance implies the need of genetic counseling.

## Figures and Tables

**Table 1:** Diagnostic protocols in NBCCS.

Family history
Past medical and dental history
Clinical examinations
Oral
Skin
Central nervous system
Head circumference
Interpupillar distance
Eyes
Genitourinary system
Cardiovascular system
Respiratory system
Skeletal system
Genetic testing
X-ray
Chest
A.P. and lateral skull
Panoramic radiograph
Cervical and thoracic spine
Hands (for pseudocysts)
Pelvic (female)

Ovarian ultrasound (female) for ovarian fibroma.

Echocardiogram (children) for cardiac fibroma.

Abbreviations: NBCCS: Nevoid Basal Cell Carcinoma Syndrome.
